# Phase Change Composite Microcapsules with Low-Dimensional Thermally Conductive Nanofillers: Preparation, Performance, and Applications

**DOI:** 10.3390/polym15061562

**Published:** 2023-03-21

**Authors:** Danni Yang, Sifan Tu, Jiandong Chen, Haichen Zhang, Wanjuan Chen, Dechao Hu, Jing Lin

**Affiliations:** 1 Guangdong Key Laboratory for Hydrogen Energy Technologies, School of Materials Science and Hydrogen Energy, Foshan University, Foshan 528000, China; 2 Key Lab of Guangdong High Property and Functional Macromolecular Materials, School of Materials Science and Engineering, South China University of Technology, Guangzhou 510640, China; 3 Research Center of Flexible Sensing Materials and Devices, School of Applied Physics and Materials, Wuyi University, Jiangmen 529020, China

**Keywords:** phase change microcapsules, low-dimensional nanofillers, thermal conductivity, thermal management applications

## Abstract

Phase change materials (PCMs) have been extensively utilized in latent thermal energy storage (TES) and thermal management systems to bridge the gap between thermal energy supply and demand in time and space, which have received unprecedented attention in the past few years. To effectively address the undesirable inherent defects of pristine PCMs such as leakage, low thermal conductivity, supercooling, and corrosion, enormous efforts have been dedicated to developing various advanced microencapsulated PCMs (MEPCMs). In particular, the low-dimensional thermally conductive nanofillers with tailorable properties promise numerous opportunities for the preparation of high-performance MEPCMs. In this review, recent advances in this field are systematically summarized to deliver the readers a comprehensive understanding of the significant influence of low-dimensional nanofillers on the properties of various MEPCMs and thus provide meaningful enlightenment for the rational design and multifunction of advanced MEPCMs. The composition and preparation strategies of MEPCMs as well as their thermal management applications are also discussed. Finally, the future perspectives and challenges of low-dimensional thermally conductive nanofillers for constructing high performance MEPCMs are outlined.

## 1. Introduction

The impending energy exhaustion crisis, severe environmental pollution, and climate change have prompted many research priorities to shift towards sustainable and renewable energy resources, such as ocean, wind, solar, and heat energy [1]. However, it remains challenging to ensure uninterrupted supply of these energy sources due to their intermittency and volatility. Therefore, developing advanced energy storage technologies is considered as a promising and efficient approach to utilize various renewable energy sources. Typically, to enhance the utilization efficiency of thermal energy, thermal energy storage (TES) technologies, including sensible heat storage (SHS), latent heat storage (LHS), and reversible thermochemical reaction, have been widely developed in the past few decades [2]. Among them, phase change materials (PCMs)-based LHS is particularly attractive owing to its large energy storage density, operational simplicity, and ability to solve the contradiction between the energy supply and demand in time and space [3]. In general, PCMs can be categorized into liquid–gas, solid–gas, solid–liquid, and solid-solid PCMs according to their phase transition states. In comparation with other types of PCMs, the solid-liquid PCMs exhibit many outstanding advantages such as low density, high energy storage capacities, chemical stability, and appropriate phase transition temperature, which has triggered extensive attention of industry and academia. Unfortunately, when the solid–liquid PCMs was employed in practical applications, several inferiors including low thermal conductivity, easy leakage, and volume change, can hinder the prosperity of TES system in various application scenarios. To address aforementioned bottlenecks, enormous efforts have been devoted to constructing shape-stabilized PCMs [4,5,6,7,8,9,10]. In particular, microencapsulated phase change materials (MEPCMs) using PCMs as capsule cores and a polymer or inorganic materials as shells have been extensively investigated, which was expected to prevent the leakage and erosion of melting PCMs during phase change process. However, it is worth noting that both polymeric and inorganic shells have their own problems. For example, although the MEPCMs with polymer shells have high chemical stability, excellent anti corrosiveness, and facile processability, their thermal conductivity is usually poor, while the MEPCMs with single inorganic materials shells are brittle and prone to leakage. Therefore, in recent years, phase-change composite microcapsules with organic-inorganic shells have gradually become the development direction and research focus in the field of MEPCMs.

In particular, the rapid development of nanomaterials in the last few years has opened many opportunities for the preparation of high-performance MEPCMs. Benefiting from their large specific surface area, excellent compatibility, and stable chemical properties, various low-dimensional thermally conductive nanofillers, such as graphene [11], boron nitride (BN) [12], black phosphorus [13] and carbon nanotubes (CNTs), were introduced in MEPCMs to endow the PCMs with higher thermal conductivity, superior stability, and heat transfer efficiency. It was found that the phase change composite microcapsules reinforced with low-dimensional thermally conductive nanofillers exhibit high packaging efficiency, excellent mechanical properties, fantastic energy storage efficiency, and promising thermal conductivity. These pioneering works have strongly promoted the practical application of MEPCMs in TES. Nowadays, MEPCMs have been widely used in many fields including thermo-regulating fabrics, building, solar energy storage system, thermal management of electronics, waste heat recovery systems, and medical transport [14,15,16,17]. However, to the best of our knowledge, there are few reviews to specifically discuss the MEPCMs containing various low-dimensional thermally conductive nanofillers.

**Figure 1:**
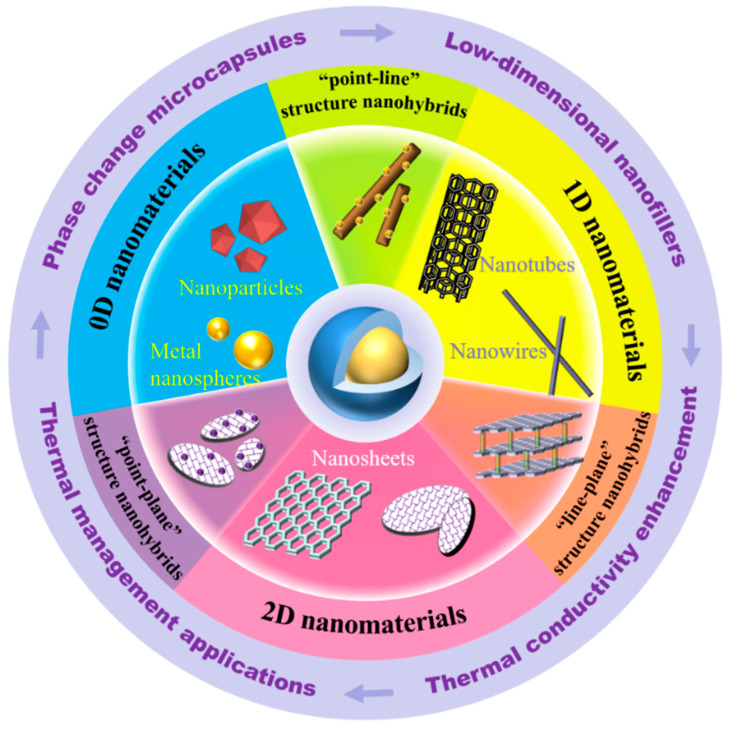
Schematic illustration of the main topics of this review regarding advanced phase change composite microcapsules with low-dimensional thermally conductive nanofillers.

Herein, this work aims to provide a comprehensive and profound understanding of the recent progress, emerging trends, and challenges of phase change composite microcapsules with low-dimensional thermally conductive nanofillers. As outlined in Figure 1, we first discuss the typical PCMs, shell materials and preparation strategies for constructing advanced MEPCMs. After that, we summarize the influence of low-dimensional nanofillers on thermal conductivity of MEPCMs as well as their thermal management application. Finally, the future research topics and challenges of low-dimensional thermally conductive nanofillers for reasonable design and construction of high performance MEPCMs are prospected. By systematically summarizing the relevant achievements and exploring their preparation–structure–property-applications relationships, we hope that this review can provide meaningful enlightenment for the architectural design and multifunction of advanced PCMs. 

## 2. Composition and Preparation of MEPCMs

### 2.1. Core Materials of MEPCMs

PCMs is a kind of typical latent heat TES material with the advantages of large heat storage density, operational simplicity, and high safety, which can control the temperature by reversibly absorbing or releasing thermal energy to the environment [18]. Thus, PCMs, as an excellent storage media, have a wide range of applications, including solar energy systems, buildings, and thermal management of vehicles battery and electronic devices [19,20,21,22,23,24,25]. Generally, PCMs can be divided into solid-solid, solid-liquid, solid-gas, and liquid-gas PCMs according to their phase transition states [26]. Although the latent heat of solid-gas and liquid-gas PCMs is larger than that of solid-liquid and solid-solid PCMs, it is difficult to be applied in engineering fields due to their great volume change in phase transition process. Besides, the solid-solid PCMs which stored heat by changing crystal form, have a relatively smaller phase transition enthalpy than solid-liquid PCMs [27]. Correspondingly, the solid-liquid PCMs have unique advantages in phase equilibrium, volume change and vapor pressure. Thus, the solid-liquid PCMs become the most significant core materials employing in phase change microcapsules [28]. When the external temperature is higher than the solid-liquid PCMs, the solid-liquid PCMs will absorb heat in the form of sensible heat, and the temperature begins to rise. When the phase transformation temperature is reached, the temperature will remain constant. This heating process can effectively store energy in the form of latent heat until it is completely converted into liquid and then raising the temperature in the form of sensible heat (and vice versa). The cooling process also has a period of releasing energy in the form of latent heat.

Solid-liquid PCMs can be further divided into organic, inorganic, and eutectic PCMs according to their composition [29]. Among them, organic PCMs have the characteristics of wide applicable temperature range and stable chemical properties, which mainly include paraffins, fatty acids, esters, and alcohols [30]. Typically, paraffins, as the most common commercial organic PCMs, contain a variety of high-purity straight chain alkanes, and their melting temperature and melting enthalpy can increase with the number of carbon atoms increasing. Compared with high-purity straight chain alkanes, paraffins are usually produced as the by-products in crude oil refining. Thus, they are widely available and inexpensive, and have higher practical application value [31,32]. Fatty acid is another organic PCMs produced from common vegetable oils and animal oils, whose cost is higher than that of paraffin. The commonly used fatty acids include stearic acid (SA) (melting point 69 °C), palmitic acid (melting point 56 °C), myristic acid (melting point 58 °C) and lauric acid (melting point 49 °C), etc. Due to their suitable phase transition temperature and high phase transition enthalpy, fatty acids are the most promising non-paraffin PCMs. Besides, sugar alcohol can also be used as PCMs, whose melting temperature and melting enthalpy are the highest among organic PCMs, so it has good application prospects in solar heating and industrial waste heat recovery. Notably, though organic PCMs have various advantages, almost all of them belong to low-temperature PCMs with a melting point lower than 220 °C [33]. Moreover, their thermal conductivity is relatively low, and usually suffer from high flammability and low thermal stability. Different from organic PCMs, the inorganic PCMs exhibit a relatively higher thermal conductivity and greater phase change enthalpy. They also have a clear melting point, excellent flame resistance, and good recyclability. Inorganic PCMs can be roughly divided into hydrated salts and molten salts. When the PCMs are hydrated salts, the solid-liquid phase change process is actually the dehydration and hydration process of hydrated salts, such as MgSO_4_·7H_2_O (melting point 49 °C), CaCl_2_·6H_2_O (melting point 33 °C), Ba(OH)_2_·8H_2_O (melting point 78 °C), Al(OH_3_)_2_·7H_2_O (melting point 75 °C). Usually, two or more hydrated salts are mixed to adjust the phase transition temperature to meet the requirements of practical applications. It is mainly suitable for medium and low temperature TES since the melting point of most hydrated salts is below 220 °C, while molten salts such as sulfate and nitrate can run at a temperature of more than 420 °C and can be used in high temperature TES applications [34,35]. Eutectic PCMs can be prepared by inorganic-inorganic, organic-organic or two kinds of mixed PCMs, which can achieve high thermal conductivity, small supercooling, and a relatively low cost through adjusting their specific proportion. Eutectic PCMs have a wide range of melting points and can be used in low, medium, and high temperature applications. Most organic-inorganic eutectic PCMs belong to low temperature PCMs, such as CO(NH_2_)_2_-NH_4_Br (melting point 80 °C), while most inorganic-inorganic eutectic PCMs belong to medium temperature PCMs with melting point between 220 °C and 420 °C [36,37]. Their advantages, disadvantages, and the melting points of typical organic, inorganic, and eutectic PCMs are summarized in Table 1.

### 2.2. Shell Materials of MEPCMs

The shell of phase change microcapsules can effectively prevent the leakage of PCMs during the phase change process. Obviously, to maintain the structural stability of microcapsules, the shell materials need to have high encapsulation efficiency, excellent thermal and chemical stability, and superior compatibility. In the past few decades, extensive efforts have been dedicated to enhance the comprehensive properties of MEPCMs by the reasonable design of shell materials [38]. Usually, these shell materials can be divided into organic, inorganic, and organic-inorganic hybrid materials according to their chemical properties. 

#### 2.2.1. Organic Shells

Organic shell materials are generally composed of natural and synthetic polymer materials which have good elasticity, toughness, excellent compactness, and stable chemical properties. Typically, melamine-formaldehyde (MF) resin and urea-formaldehyde (UF) resin are the most reported and widely used organic shell materials of microcapsules [39,40,41,42,43,44,45,46]. For example, Yu et al. designed a type of MEPCMs by using MF as the shell material and dodecanol as the core material via in-situ polymerization, achieving the highest phase transition enthalpy of 187.5 J/g and a high encapsulation efficiency of 93.1% [45]. Compared with MF, UF has higher phase transition latent heat and relatively poor thermal resistance [47,48,49,50,51,52,53]. Thus, Tohmura et al. developed melamine-urea-formaldehyde resin (MUF) by the simultaneous reaction of cyanuric acid and urea with formaldehyde to effectively improve the intermolecular force, thereby improving its thermal stability [53]. Besides, polymethylmethacrylate (PMMA) is also a common shell material for MEPCMs in recent years due to its environmental stability, and low easy processing cost [54,55,56,57,58]. However, the residual small molecules (such as formaldehyde, acrylate, etc.) of the above resins pose a threat to the environment and human health. Therefore, more and more studies have been devoted to the development of polyurea or polyurethane (PU) encapsulated MEPCMs [59,60,61]. In particular, the initial reaction rate between diisocyanate and polyol is relatively lower, and the elasticity of MEPCMs shell can be improved by adjusting the soft segment structure of PU, which is very beneficial to the mechanical properties and compactness of MEPCMs [62]. Therefore, in the previous study, our team has realized the microencapsulation of methyl laurate with PU shell through interfacial polymerization [63,64,65]. In addition, some polymers including polystyrene (PS) [66,67,68,69,70] and starch [71], etc are also used as organic shell materials to prepare MEPCMs. However, it is noting that most organic materials face the challenges of high flammability and low thermal conductivity.

#### 2.2.2. Inorganic Shells

Compared with the organic polymers, the inorganic shell materials have higher thermal conductivity and superior mechanical strength, which can not only improve the durability and reliability of the microcapsules, but also improve the heat transfer performance of MEPCMs. Therefore, the use of inorganic materials as microcapsules shells has become another significant trend [38]. Common inorganic shell materials include silica oxide (SiO_2_) [72,73,74,75,76,77,78,79,80,81,82,83,84,85,86,87,88], calcium carbonate (CaCO_3_) [89,90,91,92,93], graphene [94,95,96], titanium dioxide (TiO_2_) [97,98,99,100], etc. Among them, SiO_2_ is a commonly used shell due to its wide source, low cost, and mature preparation method. For example, Liang et al. developed n-octadecane-based MEPCMs with silica shell by interfacial polymerization, and the phase change enthalpy and encapsulation rate of the microcapsules were as high as 109.5 J/g and 51.5%, respectively [73]. Besides, the graphene with superb intrinsic thermal conductivity can significantly improve the thermal conductivity of MEPCMs and provide a new material for thermal energy management [96]. In addition, metal oxides have also been widely used as an inorganic shell, including TiO_2_, Al_2_O_3_, Fe_3_O_4_, and ZnO, etc [101]. For example, Liu et al. synthesized n-eicosane MEPCMs with TiO_2_ as shell by interfacial polymerization, and found that the obtained microcapsules not only had durability and highly stable shape, but also exhibited superior antibacterial function, showing great potential in medical applications [99]. However, it is undeniable that the inorganic shell materials inevitably suffer from the disadvantage of poor stability and brittleness.

#### 2.2.3. Organic-Inorganic Hybrid Shells

Organic-inorganic hybrid shells can combine the high inherent thermal conductivity of inorganic materials and the toughness of polymer shells, and achieve a high thermal conductivity, permeability resistance and thermal stability of MEPCMs. Therefore, the organic-inorganic hybrid shell exhibit promising potential in next-generation shell materials of MEPCMs [38,102]. It can be expected that the incorporation of inorganic nanofillers in organic shells can endow MEPCMs with high thermal conductivity and some unique properties [103,104,105,106,107,108]. Typically, Pickering emulsion polymerization is a classical method to obtained the polymer/inorganic hybrid shells. For example, Yin et al. prepared n-hexadecanol MEPCMs using MF-SiO_2_ as the hybrid shell by Pickering emulsion polymerization [103]. The SiO_2_ particles in the shell significantly improved the mechanical strength and thermal reliability of the MEPCMs, and received a high phase change enthalpy of 163.76 J/g. Furthermore, Zhang et al. synthesized a unique graphene oxide (GO)/polyaniline (PANI) hybrid shell through emulsion polymerization, which combined the excellent barrier properties of GO with the anti-corrosion function of PANI. The results show that the MEPCMs have strong solvent resistance to organic solvents, and the MEPCMs are dispersed in waterborne epoxy resin, which can prepare intelligent coatings with dual functions of anti-corrosion and self-healing [109]. In short, the MEPCMs with polymer-inorganic hybrid shells have become the current research focus of phase change microcapsules, which hold great potential in thermal management applications. Table 2 summarizes the phase change enthalpy and encapsulation rate of MEPCMs encapsulated by some representative shell materials.

### 2.3. Preparation Strategies of MEPCMs

The microencapsulation of PCMs with various shell materials can not only effectively solve the leakage problem during the phase change process, but also improve the thermal conductivity and other properties of PCMs, which shows a good development prospect in many applications. Most of MEPCMs are spherical with a particle size from 1 to 1000 μm, and their mass of PCMs cores vary from 20% to 95% in the total mass of MEPCMs [110]. Typical preparation strategies of the MEPCMs were described as follows. It can be mainly divided into physical method, chemical method, and physical-chemical method according to the synthesis mechanism. 

#### 2.3.1. Physical Method

**Figure 2:**
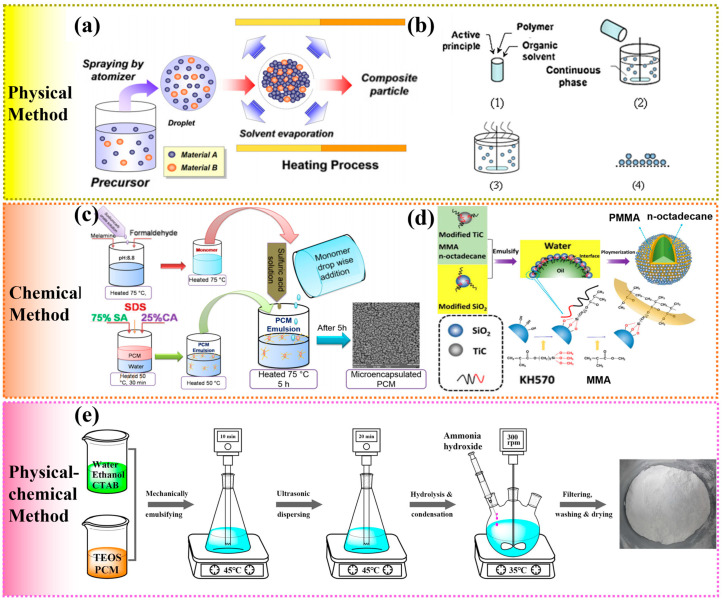
(**a**) Schematic diagram of the mechanism for preparing microcapsules using spray drying. Reprinted with permission from [111]. Copyright 2011 Elsevier; (**b**) Basic steps of microencapsulation by solvent evaporation. Reprinted with permission from [115]. Copyright 2008 Elsevier; (**c**) Schematic presentation of stepwise preparation of MEPCMs by in-situ polymerization. Reprinted with permission from [117]. Copyright 2020 Wiley; (**d**) Schematic preparation of microcapsules with a polymer-SiO_2_/TiC hybrid shell by Pickering emulsion polymerization. Reprinted with permission from [118]. Copyright 2018 Elsevier; (**e**) Schematic preparation process of MEPCMs by sol-gel method. Reprinted with permission from [119]. Copyright 2020 Elsevier.

In general, the physical methods for preparing MEPCMs mainly include spray drying and solvent evaporation [111,112]. The spray drying method is that the core material and the shell material are co-dissolved in a solvent, and the mixture is sent into a heating chamber in the form of small droplets for heating. During the heating process, the solvent will evaporate, and finally the microcapsules are separated. For example, Borregro et al. microencapsulated the paraffin by spray drying, and the encapsulation efficiency reached 63%. Besides, they found that the properties of these MEPCMs were closely related to where they were collected in the spray dryer, and the MEPCMs was highly stable and reversible even after 3000 cycles [112]. The schematic diagram of the mechanism is illustrated in Figure 2a. Overall, the spray drying method is relatively simple to operate and has a high production efficiency, but it is not suitable for inorganic PCMs [113]. Another physical synthesis method is the solvent evaporation method [114]. Specifically, the polymer shell materials were dissolved in volatile solvent, and then adding PCMs into above solution to form emulsion, finally the shell is formed on the droplet surface by evaporating the solvent. Figure 2b shows the preparation route of MEPCMs by solvent evaporation [115]. More importantly, in comparation with the spray drying method, it can be used to synthesis MEPCMs with inorganic materials. However, this synthesis method still suffers from the low encapsulation efficiency [116].

#### 2.3.2. Chemical Method

Chemical method is the most widely used method to prepare MEPCMs. It usually uses free radical polymerization to form oil-in-water or water-in-oil emulsions, and reacts on the oil/water (O/W) interface to form shell materials. The representative and commonly used chemical methods include in-situ polymerization, interfacial polymerization, suspension polymerization and emulsion polymerization. Typically, the in-situ polymerization method is to emulsify the core material to form oil-in-water emulsion droplets under the action of emulsifier, and then the obtained O/W emulsion droplets were catalytically polymerized with monomers or prepolymers under certain conditions to form a polymer shell, and finally cured to form MEPCMs (Figure 2c). Generally, most of MEPCMs with MF and UF shells are synthesized by in-situ polymerization. Notably, this synthesis method can achieve the synergetic integration of high encapsulation efficiency and uniform coating, but the operation process is relatively complex and may cause some environmental pollution [117]. Another chemical method for preparing MEPCMs is interfacial polymerization. In the interfacial polymerization, the core material and hydrophobic monomer are used as the oil phase, while the emulsifier and deionized water serve as the water phase, which are emulsified to form an oil-in-water emulsion, and finally polymerize at the O/W interface to form a shell under appropriate conditions. Zhang et al. synthesized a n-octadecane-based MEPCMs with a polyurea shell by interfacial polymerization. It was found that when the core-shell ratio was 7/3, the MEPCMs exhibited better phase transition performance, better permeation resistance and higher encapsulation efficiency [120]. In general, interfacial polymerization is easy to operate and low cost, but the reaction speed is often too fast, resulting in the properties of final product is more difficult to control [121]. Besides, suspension polymerization is to mix the PCMs, reaction monomers and initiator as the oil phase, which is then suspended in the water phase to form emulsion droplets under the action of surfactants, finally polymerize to form MEPCMs [122]. Typical MEPCMs with organic shell materials such as PMMA and PS are usually prepared by suspension polymerization. Compared with above polymerization methods, the suspension polymerization usually has a higher encapsulation rate and strong thermal regulation ability, but the production cost is also relatively higher [116]. In addition, the emulsion polymerization method is to mix the PCMs with reaction monomers, and add emulsifiers to form the O/W emulsions, finally initiate polymerization to form microcapsules under the action of initiator [123,124]. Particularly, Pickering emulsion polymerization has been widely applied to prepare MEPCMs in recent years due to the superior mechanical properties and thermal stability of as-obtained MEPCMs. Moreover, Pickering emulsion polymerization is more environmentally friendly and easy to obtain polymer/inorganic hybrid shells [116,125]. As shown in Figure 2d, Wang et al. prepared a MEPCMs with Pickering emulsion polymerization by introducing modified SiO_2_ and TiC nanoparticles as emulsifiers [118]. The obtained MEPCMs exhibited well-defined core-shell structure, and have good heat storage and release properties, superior thermal stability, and enhanced thermal conductivity. So far, Pickering emulsion polymerization has become an important part of the methods for preparing of advanced MEPCMs due to its simplicity and economy. 

#### 2.3.3. Physical-Chemical Method

Physical-chemical methods are usually used to prepare microcapsules under external force and chemical reaction by combining physical processes such as heating and cooling with chemical processes such as crosslinking and condensation. Coacervation is a typical and commonly used physical-chemical method, which can be further divided into single coacervation and complex coacervation method [126,127]. For example, complex coacervation method is to form emulsion by mixing PCMs with a polymer, then emulsifying with another polymer to form stable emulsion, and finally condensing to form MEPCMs. Sol-gel method is to hydrolyze the reaction monomer to form sol solution, and then mix it with the PCMs to form emulsion [128,129]. Under certain reaction conditions, the gel shell is generated around the PCMs droplet through polycondensation reaction (Figure 2e) [119]. 

In summary, there are many encapsulation methods that can be used to prepare MEPCMs. However, as shown in Table 3, different preparation methods all have some advantages and disadvantages. For example, although physical methods are simpler and more economical, the prepared MEPCMs are usually agglomerated together. Moreover, Pickering emulsion polymerization is particularly suitable for the preparation of MEPCMs with organic-inorganic hybrid shells, which is of great significance for achieving the enhanced thermal conductivity and functionalization of MEPCMs, but still faces some challenges for large-scale implementation. Therefore, in practical applications, the selection of an encapsulation method not only depends on the performance and parameters of MEPCMs (e.g., size, size distribution, chemical stability, and thermal stability), but also is inextricably linked to the polymerization operability, cost, etc.

## 3. Thermal Conductivity Enhancement of PCMs with Low-Dimensional Nanofillers

PCMs have high heat storage density and relatively stable temperature change. However, pristine PCMs generally suffer from low intrinsic thermal conductivity, which limits the thermal transfer efficiency of the system and is one of the long-standing bottlenecks restricting their practical applications [130,131]. Thus, in the past years, to remedy this defect, low-dimensional nanofillers are commonly used to improve the thermal conductivity of PCMs by virtue of their ultrahigh intrinsic thermal conductivity and small size effect [132]. In this section, the influences of various low-dimensional nanofillers on the thermal conductivity of PCMs were systematically reviewed. 

### 3.1. Zero-Dimensional Nanofillers

**Figure 3:**
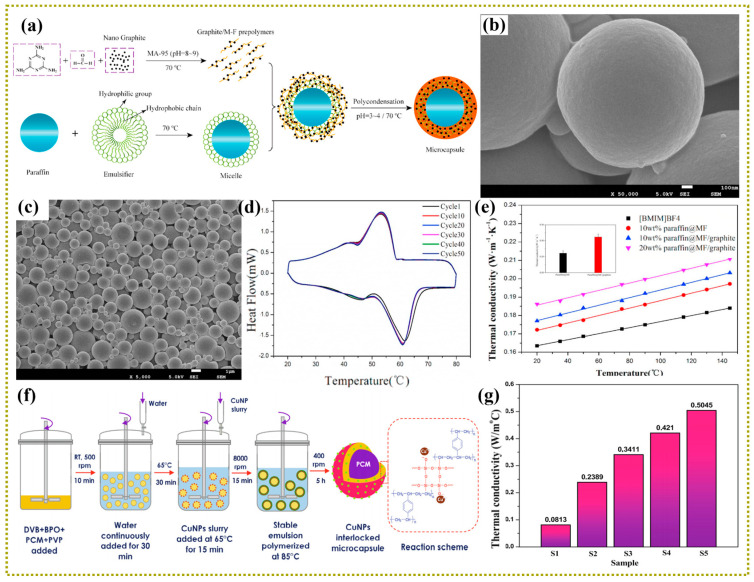
(**a**) Schematic preparation of the paraffin@MF/graphite MEPCMs, (**b**) SEM images of paraffifin@MF/graphite MEPCMs, (**c**) SEM image and (**d**) DSC curves of the paraffifin@MF/graphite MEPCMs after 50 heating−cooling cycles, (**e**) Thermal conductivity of the slurries containing different MEPCMs. Reprinted with permission from [134]. Copyright 2017 Elsevier; (**f**) Schematic diagram of preparing CuNPs interlocked MEPCMs through suspension polymerization, (**g**) Thermal conductivity of MEPCMs fabricated with different dosages of CuNPs. Reprinted with permission from [135]. Copyright 2021 Elsevier.

Zero-dimensional (0D) nanofillers (e.g., silver nanoparticles (AgNPs), nanodiamond, copper nanoparticles (CuNPs), Fe_3_O_4_) have high intrinsic thermal conductivity, which have been widely utilized to enhance the thermal conductivity of PCMs [133]. In general, the solid PCMs transfers heat by inter-lattice vibrations in the crystalline state, while in the melting state, the heat transfer of PCMs is mainly due to the energy transfer from the collision between matrix molecules by Brownian motion. The incorporation of 0D nanofillers can increase the collisions probability between adjacent nanoparticles or between nanoparticles and matrix molecules. For instance, Liu et al. fabricated MEPCMs with the paraffin as core material and the graphite nanoparticles modified MF as shell by in-situ polymerization method (Figure 3a) [134]. As shown in Figure 3b, it was found that the surface of paraffin@MF/graphite composite microcapsules seemed to be smoother than that of the paraffin@MF microcapsules, which may be due to the added graphite nanoparticles preventing the MF prepolymer drastically flocculating on the surfaces of MEPCMs. The obtained results showed that the GO was in favor of stabilizing the MEPCMs dispersed phase change slurry. Figure 3c,d shows the scanning electron microscope (SEM) images and differential scanning calorimetry (DSC) curves of paraffin@MF/graphite after 50 cold-heat cycles. The results show that the paraffin@MF/graphite composite microcapsules have extraordinary morphological stability, high encapsulation rate of 51.1%, and superior thermal conductivity of 0.312 W/(m·K) (Figure 3e). Furthermore, Parvate et al. successfully prepared a novel functionalized CuNPs interlocking polydivinylbenzene hexadecane MEPCMs by suspension polymerization technology (Figure 3f), which showed excellent thermal reliability and thermal conductivity of 0.5045 W/(m·K) (Figure 3g) [135]. The as-prepared CuNPs/MEPCMs are particularly suitable for the storage and packaging of food for the extended thermal buffering duration. Moreover, Wang et al. designed new MEPCMs with n-octadecane as core and poly (melamine-formaldehyde)/silicon carbide (PMF/SiC) as shell by in-situ polymerization [136]. Compared with PMF microcapsules, the thermal conductivity of n-octadecane@PMF/SiC composite microcapsules increased by 60.34% after incorporating 7% nano-SiC. The prepared microcapsules efficiently absorbed near-infrared light and displayed supernormal photothermal conversion performance under light radiation. Additionally, Zhu et al. successfully modified the silica-microencapsulated MEPCMs with polydopamine (PDA) and silver layer [137]. As the concentration of AgNO_3_ increases, more 0D Ag nanoparticles are deposited on the surface of MEPCMs until they form a continuous silver layer. The apparent thermal conductivity of silver-coated MEPCMs is significantly increased to 1.346 W/(m·K), indicating that silver-coated PCMs are promising to apply in TES system, especially for electronic devices requiring rapid heat transfer. In addition, the researchers propose that the thermal conductivity of PCMs can also be enhanced by adding metal oxide nanoparticles with high thermal conductivity [138]. However, it is noting that the low aspect ratio characteristic of 0D nanoparticles usually leads to large heterogeneous interfaces, which is detrimental to form thermal conduction pathway. Moreover, the nanosized particles are easy to result in agglomeration and poor dispersion, which is also not conducive to the synthesis of advanced MEPCMs.

### 3.2. One-Dimensional Nanofillers

**Figure 4:**
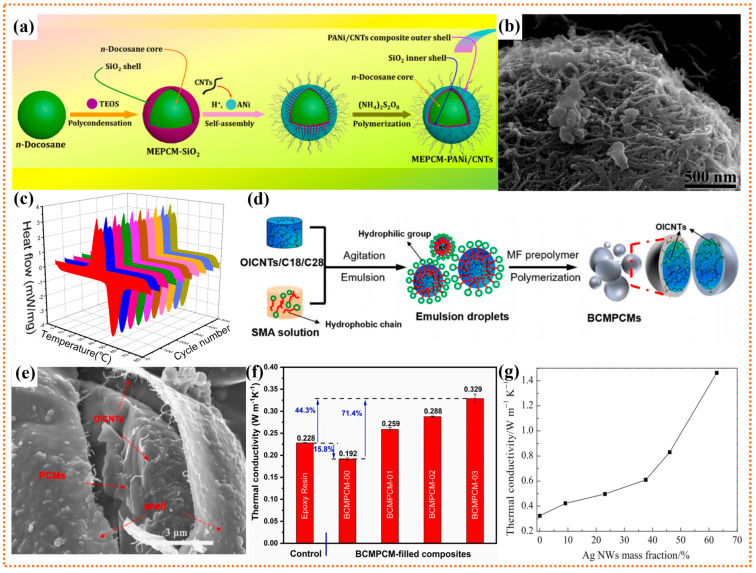
(**a**) Scheme of synthetic route for the n-docosane@SiO_2_/PANi/CNTs MEPCMs, (**b**) SEM micrographs of n-docosane@SiO_2_/PANi/CNTs MEPCMs, (**c**) DSC thermograms of the MEPCMs after thermal cycling treatment at every 50th cycles. Reprinted with permission from [141]. Copyright 2020 Elsevier; (**d**) The fabrication process of BCMPCMs with OICNTs, (**e**) SEM micrographs of the fractured microcapsules of BCMPCM, (**f**) Comparison of the heat transportation capabilities of different BCPCMs in epoxy composites. Reprinted with permission from [142]. Copyright 2020 Elsevier; (**g**) Thermal conductivity of the composite PCMs containing AgNWs. Reprinted with permission from [144]. Copyright 2010 Springer Nature.

In comparation with the point contact of 0D nanofillers, the one-dimensional (1D) nanofillers are easier to form highly efficient thermal conduction pathways under the equivalent filler loadings. Thus, various 1D nanofillers have also been used in PCMs to improve their thermal conductivity. Typically, CNTs sparked the research interest of PCMs field due to its low density, high aspect ratio, outstanding mechanical properties, and excellent thermal conductivity [139]. Arshad et al. explored the influence of multi-walled CNTs (MWCNTs) on PCMs [140]. The results showed that the thermal conductivity of PCMs mixed with MWCNTs is 61.2% higher than that of pure PCMs, and the value of thermal conductivity and phase-change enthalpy reached 0.3551 W/(m·K) and 248.22 J/g, respectively, which proves that the addition of CNTs is more conductive to meeting the requirements of microelectronic thermal management. To further improve the thermal conductivity of MEPCMs, Sun et al. designed and manufactured a novel layered MEPCMs functionalized with PANi/CNTs, and the obtained n-docosane@SiO_2_/PANi/CNTs MEPCMs exhibited extraordinary temperature self-regulation capability and high thermal conductivity of 0.846 W/(m·K) (Figure 4a) [141]. SEM images show that CNTs are densely distributed on the surface of microcapsule (Figure 4b). As observed in Figure 4c, the DSC curves exhibit almost identical profiles with a high coincidence in crystallization and melting peaks at every 50th cycle number. However, due to the poor compatibility between CNTs and PCMs, it is still challenging to achieve the uniform distribution of CNTs in microcapsules, which inevitably reduces the modification effect of CNTs on MEPCMs. In order to strengthen the interfacial compatibility between CNTs and PCMs, Meng et al. used octecyl isocyanate (OI) modified CNTs to developed a new type of thermal conductivity enhanced binary-core MEPCMs (BCMPCMs) with n-octadecane/n-octacosane (C18/C28) binary core through in-situ polymerization (Figure 4d) [142]. The results showed that the thermal conductivity of OICNT-enhanced BCMPCM/epoxy resin composites exhibited a 71.4% increase over that of the pure BCMPCM/epoxy resin composite. Figure 4e shows the SEM image of broken MEPCMs. It was found that many worm-like CNTs were evenly dispersed within the core of BCMPCMs, and the thermal conductivity of BCMPCMs was increased by 71.4% to 0.329 W/(m·K) (Figure 4f). Furthermore, infrared thermal imaging analysis shows that the BCMPCMs has two different temperature buffers and significant temperature drop, which indicates that the MEPCMs has a supernormal potential for applications in the field of TES and temperature regulation. Moreover, it was found that functionalized multi-walled carbon nanotubes (f-MWCNTs) can effectively reduce the supercooling of paraffin wax. Tang et al. synthesized a paraffin/f-MWCNTs phase change composite by carboxylating f-MWCNTs with mixed acid of H_2_SO_4_ and HNO_3_ and then salt-forming reaction with n-octadecylamine [143]. DSC analysis showed that the addition of f-MWCNTs reduced the supercooling of paraffin, mainly due to the well-dispersed f-MWCNTs serving as nuclei to promote the heterogeneous nucleation and crystallization process of paraffin. Additionally, silver nanowire (AgNWs) is another 1D nanostructured material, which can significantly improve the thermal conductivity of PCMs by virtue of its high aspect ratio and low interfacial thermal resistance. Zeng et al. fabricated a composite PCMs containing AgNWs, achieving a high thermal conductivity (1.46 W/(m·K)) and phase change enthalpy (76.5 J/g) (Figure 4g) [144]. The above results suggest that AgNWs may be a promising candidate for enhancing the thermal conductivity of organic PCMs. However, AgNWs are susceptible to oxidation and corrosion, which may lead to a reduction in heat transfer efficiency. Additionally, the MEPCMs filled with metallic fillers and CNTs are also restricted in the field of electronic devices that require high electrical insulation.

### 3.3. Two-Dimensional Nanofillers

Since the discovery of graphene in 2004, two-dimensional (2D) nanofillers have attracted a lot of attention from researchers in many fields. In particular, many 2D nanomaterials (e.g., graphene, BN) have a large specific surface area and superhigh intrinsic thermal conductivity, which can effectively enhance the thermal conductivity of PCMs and prevent their leakage [145,146]. Moreover, the application of 2D nanofillers in MEPCMs can also endow the PCMs with versatility, promoting their wider applications in many fields [147,148,149,150,151]. 

**Figure 5:**
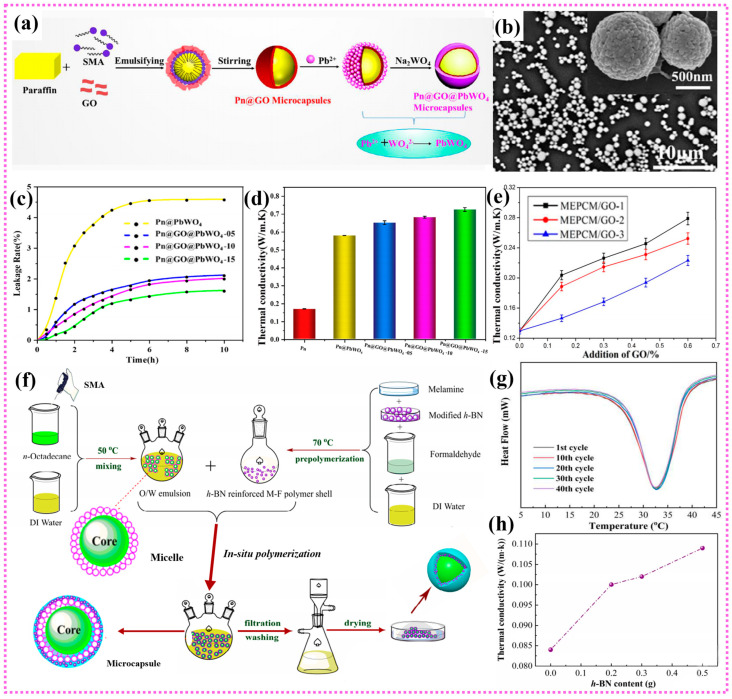
(**a**) Schematic of preparation for Pn@GO@PbWO_4_ microcapsules, (**b**) SEM images of Pn@GO@PbWO_4_ microcapsules, (**c**) Leakage rate curves of microcapsules, (**d**) Thermal conductivity of Pn and the microcapsules with different contents of GO. Reprinted with permission from [156]. Copyright 2021 Wiley; (**e**) Effect of the oxidation degree on thermal conductivity of MEPCMs. Reprinted with permission from [157]. Copyright 2018 Elsevier; (**f**) Schematic illustration of the synthesis of n-octadecane@BN/MF microcapsules, (**g**) DSC curves of the n-octadecane@BN/MF microcapsules after experiencing heating-cooling cycles, (**h**) Thermal conductivity of the composites with different h-BN content. Reprinted with permission from [12]. Copyright 2020 Elsevier.

Graphene, the most investigated 2D carbon allotrope, is composed of a monolayer of hexagonally arranged sp^2^-bonded carbon atoms. Recently, benefiting from its ultrahigh intrinsic thermal conductivity (~5300 W/(m·K)) and prominent mechanical properties, graphene and graphene derivatives have gained a lot of research interest in the field of thermal management [152,153]. Particularly, the combination of graphene nanosheets and PCMs can significantly improve the thermal conductivity of energy storage systems, thus making the idea of industrial energy management system more realistic [154]. For instance, Zhang et al. designed a n-hexadecane based MEPCMs with PS/GO double-shell by Pickering emulsion method, showing high sealing rate and thermal stability [108]. Similarly, SA/graphene MEPCMs were also fabricated by Dao et al. via the same method, and the results showed that the graphene shell effectively improved the thermal conductivity of MEPCMs (0.352 W/(m·K)) [155]. Moreover, the graphene shell with outstanding barrier property can serve as the protective layers for the SA cores and endow the MEPCMs with excellent thermal stability. Wei et al. developed a new multifunctional MEPCMs with paraffin as the core and GO/PbWO_4_ as the double shell [156]. The preparation diagram and SEM images of Pn@GO/PbWO_4_ microcapsule are shown in Figure 5a,b. Compared with pure MEPCMs, PbWO_4_ and GO can increase the leakage resistance rate of MEPCMs by 67.27% (Figure 5c). The study also showed that the addition of GO effectively improved the encapsulation efficiency and the thermal conductivity of MEPCMs (0.735 W/(m·K)) (Figure 5d). More importantly, the obtained MEPCMs exhibit excellent superhydrophobic properties and gamma radiation shielding ability due to the combination of GO and PbWO_4_ double shell. In addition, Liu et al. also investigated the effect of oxidation degree of GO on the thermal properties of MEPCMs, and synthesized GO modified dodecanol/MF composite microcapsules with different oxidation degrees [157]. When the amount of GO was about 0.6 wt%, the thermal conductivity of MEPCMs with the lowest oxidation degree of GO increased by 115%, which greatly improves the energy storage efficiency (Figure 5e). However, there are still exist challenges in the pursuit of good compatibility between inorganic particles with the core or shell materials in MEPCMs. Therefore, Chen et al. developed a novel octadecamine-grafted graphene oxide (GO-ODA) modified MEPCMs by in-situ polymerization [158]. The alkylated GO is highly compatible with the core material. The addition of GO-ODA not only made the MEPCMs exhibit good thermal cycle stability and excellent thermal conductivity during the phase transition process, but also promoted the crystallization of n-octadecane, resulting in a significant decrease in the degree of supercooling. Even so, the addition of graphene will inevitably lead to an increase in the electrical conductivity of target composites, which makes it difficult to meet the electrical insulation requirements in some microelectronic fields. Fortunately, different from the graphene, hexagonal boron nitride (h-BN) have alternating boron and nitrogen atoms that replace carbon atoms in the hexagonal honeycomb structure, whose ionic nature of B-N bond endow BNNS with outstanding electrical insulation [159,160,161,162]. Therefore, the BNNS have been widely utilized in PCMs to develop advanced MEPCMs with synergetic high thermal conductivity and electrical insulation [163,164,165,166,167]. For example, Xia et al. reported a novel n-octadecane@BN/MF MEPCMs by using BN to effectively enhance the thermal conductivity of MF shell (Figure 5f) [12]. As shown in Figure 5g, the DSC curves of MEPCMs remained highly overlapping even after 40 cycles, while the melting and crystallization temperatures are also very consistent, indicating that the synthesized MEPCMs has excellent thermal-cold cycling reliability. This study also found that the thermal conductivity of MEPCMs increased with the BN content increasing (Figure 5h). Besides, Wang et al. prepared lauric acid/modified BN nanosheet-sodium sulfate composite PCMs by vacuum impregnation method [168]. In order to ensure the uniformity and stability of h-BN suspension and improve the thermal conductivity of PCMs, h-BN was modified by isopropanol and ultrasonic stripping. The experimental results show that the thermal conductivity of PCMs can reach 0.744 W/(m·K), which is 196.4% higher than that of pure lauric acid. Furthermore, Liao et al. designed a form-stable phase change composite with high thermal conductivity and adjustable heat management [169]. By adding SA@SiO_2_ MEPCMs and surface-modified boron nitride (m-BN), the thermal conductivity of phase change composite can be elevated up to 0.506 W/(m·K). Among them, it is worth mentioning that the m-BN is fabricated by plasma treatment and then grafted with silane coupling agent, which enhances the interface affinity between BN platelets and polymer matrix. In particular, different from conventional chemical treatment, the plasma process exhibited high efficiency and no generation of liquid waste. These significant studies indicate that the application of BNNS in MEPCMs is expected to open new opportunities for enhancing their thermal conductivity and achieve extensive application in electronic devices to prolong their service life and operation reliability. In addition to graphene and BN, other 2D materials such as black phosphorus and MXene, also present favorable application prospects in MEPCMs. For instance, Huang et al. studied and prepared a MEPCMs with black phosphorus sheet (BPs) modified PMMA as the shell [13]. It is found that the mBPs-MEPCM composites exhibited a high latent heat of more than 180 kJ/kg, and the solar light absorption was largely enhanced after the BPs incorporation. Besides, the unique two-dimensional planar structure of MXene can lead to promising thermophysical properties of MEPCMs and improve their photothermal conversion efficiency [170]. Aslfattah et al. successfully synthesized a novel phase change composite using MXene (Ti_3_C_2_) as additive and demonstrated a 16% increase in thermal conductivity [171]. It was found that the melting point of the synthesized nanocomposite was slightly increased with the MXene loading increasing. Moreover, with the rapid development of nanotechnology, various novel 2D nanofillers would be gradually discovered, which may further promote the preparation of high-performance MEPCMs with high thermal conductivity and other functionality.

### 3.4. Low-Dimensional Hybrid Nanofillers

Generally, owing to the physical stacking and severe aggregation, it is still challenging to significantly enhance the thermal conductivity of MEPCMs by adding a single kind of low-dimensional nanofillers. Fortunately, the thermally conductive low-dimensional hybrid nanofillers can synergistically disperse and optimize the thermal conductivity network in MEPCMs, showing superior thermal conductivity than a single filler. In addition, low-dimensional hybrid nanofillers can give full play to the advantages of various thermally conductive nanofillers and endow the MEPCMs with special functionality. Consequently, the MEPCMs filled with different low-dimensional hybrid nanofillers have aroused tremendous interest. These low-dimensional hybrid nanofillers can be mainly divided into “point-line” structure hybrid nanofillers, “point-plane” structure hybrid nanofillers and “line-plane” structure hybrid nanofillers based on their nanostructure and contact types of nanofillers [172].

**Figure 6:**
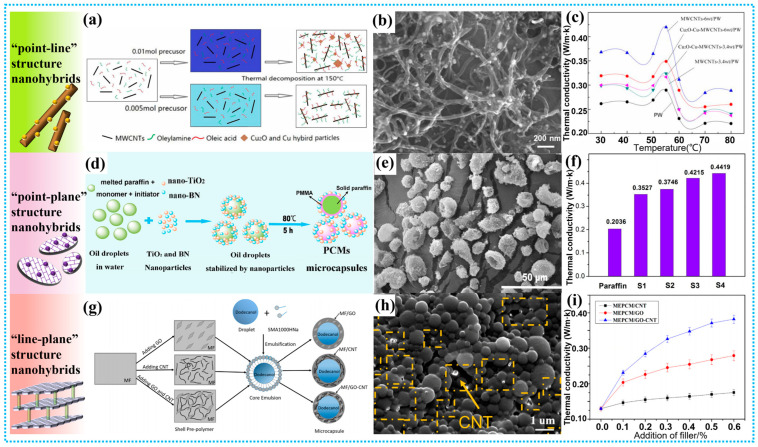
(**a**) Synthesis of Cu_2_O-Cu-MWCNTs/paraffin composites; (**b**) SEM photographs of Cu_2_O-Cu-MWCNTs composite; (**c**) Dependence of the thermal conductivity of pure paraffin, MWCNTs/paraffin and Cu_2_O-Cu-MWCNTs/paraffin composites on the temperature. Reprinted with permission from [173]. Copyright 2017 Elsevier; (**d**) Schematic fabrication of MEPCMs with paraffin core and PMMA/BN/TiO_2_ hybrid shell, (**e**) SEM images of MEPCMs prepared with PMMA/BN/TiO_2_ shell, **(f)** Thermal conductivity of pure paraffin and MEPCMs with different dosages of BN/TiO_2_ hybrid nanoparticles. Reprinted with permission from [104]. Copyright 2017 American Chemical Society; (**g**) Schematic diagrams of MEPCM with GO or CNT, (**h**) SEM images of MEPCM/GO-CNT, (**i**)Thermal conductivity of the microcapsules with different fillers. Reprinted with permission from [175]. Copyright 2019 Elsevier.

As the name implies, the “point-line” hybrid nanofillers were synthesized by combining the 0D nanomaterials and 1D nanomaterials, which is conductive to forming effective thermal conductivity pathway and reducing the interfacial thermal resistance. For example, Xu et al. prepared a new paraffin-based light-thermal conversion PCMs via introducing Cu_2_O-Cu-MWCNTs hybrid nanofillers (Figure 6a) [173]. Figure 6b shows the SEM photographs of Cu_2_O-Cu-MWCNTs hybrid nanofillers. Benefiting from the high light absorption ability of Cu_2_O and the reduced interfacial heat resistance between MWCNTs and paraffin, the obtained Cu_2_O-Cu-MWCNTs/paraffin composites exhibit excellent photothermal conversion performance, high thermal conductivity, and superior latent heat performance (Figure 6c). Furthermore, Rabady et al. studied the thermal conductivity of sodium thiosulfate pentahydrate PCMs with CNTs-graphite hybrid nanofillers [174]. The results show that their thermal conductivity can be effectively improved by increasing the mass fraction of hybrid nanofillers, and their thermal conductivity can reach 4.031 W/(m·K). Moreover, the addition of nanofillers in sodium thiosulfate pentahydrate can effectively reduce the charging and discharging time at the melting point, and at the same time, the supercooling of sodium thiosulfate hydrate has been greatly refined, thus promoting their wide application.

The “point-plane” structure hybrid nanofillers fabricated with 0D nanomaterials and 2D nanomaterials is another kind of hybrid nanofillers to enhance the thermal conductivity of MEPCMs. In “point-plane” hybrid nanofillers, the 0D nanoparticles can prevent serious stacking of 2D materials and optimize their thermal conductivity path [176]. For example, Sun et al. encapsulated paraffin in a rigid PMMA/BN/TiO_2_ shell, and SEM images showed that the synthesized MEPCMs were nearly spherical in size of 10–20 µm, and BN/TiO_2_ hybrid nanoparticles were tightly embedded on the surface of the microcapsules (Figure 6d,e) [104]. The thermogravimetric analysis (TGA) results show that the PMMA/BN/TiO_2_ composite shell makes the paraffin-based microcapsule have excellent thermal stability. Compared with pure paraffin, the thermal conductivity of MEPCMs was also significantly increased by 117.0% to 0.4215 W/(m·K) (Figure 6f). After 100 cycles of cold-heat cycling tests, the composite microcapsules still showed similar phase transition performance as before. Besides, in order to improve the thermal conductivity of clay mineral-based PCMs, Zhan et al. prepared SA/two-dimensional montmorillonite/Ag nanoparticles (2D-MT/SA/AgNPs) composite microcapsule. Compared with pure SA microcapsules, after adding AgNPs/2D-MT hybrid filler, the thermal conductivity of SA@2D-Mt/AgNPs microcapsules increased by 229.3% to 0.8223 W/(m·K) [177]. Moreover, Yuan et al. using SiO_2_/GO shells to significantly improve the thermal conductivity of paraffin-based MEPCMs to 1.162 W/(m·K) [178]. The SEM images showed that the surface of paraffin@SiO_2_/GO microcapsules was smoother than that of paraffin@SiO_2_, which may be related to the strong bonding effect of GO.

Moreover, it was found that 1D nanomaterials connecting 2D nanosheets can form an effective thermal conduction pathway in the polymer matrix and provides a fast transport channel for phonons. Therefore, the thermal conductivity of PCMs can be effectively improved by adding the “line-plane” structure hybrid nanofillers. Typically, Liu et al. prepared dodecanol-based MEPCMs with the GO-CNTs hybrid fillers modified MF shells by in-situ polymerization (Figure 6g) [175]. As observed in Figure 6h, all of the microcapsules have a regular spherical structure and their diameters is between 300~800 nm. Although some CNTs extend out of the microcapsule, the morphology of MEPCMs containing GO or CNTs is similar to that of MEPCMs without any filler, which indicates that adding GO or CNTs to the shell has little influence on their final morphology. The results also show that when the hybrid filler loading is 0.6 wt%, the thermal conductivity of MEPCMs reaches 0.3821 W/(m·K) (Figure 6i), which is 2.95 times that of the microcapsules without any thermally conductive fillers. Besides, the outstanding thermal and photo-thermal conversion properties made the MEPCMs slurry a potential fluid in direct absorption solar collectors. Additionally, Arshad et al. found that the combination of graphene nanoplatelets (GNPs) and MWCNTs nanofillers can achieve a 96% enhancement of thermal conductivity than pure PCMs and an optimum phase-change enthalpy of 245.18 J/g [140]. Table 4 summarizes the thermal conductivity of various typical MEPCMs with low-dimensional nanofillers. Based on above discussion, it can be obviously found that the low-dimensional hybrid nanofillers can significantly enhance the thermal properties of MEPCMs and widen their practical applications.

## 4. Thermal Management Applications

Due to their excellent physical and chemical properties, the aforementioned MEPCMs filled with low dimensional nanofillers have become significant candidates in many thermal management applications, such as in electronic devices, textiles, energy-saving buildings, solar energy, industrial heat recovery, greenhouse agriculture, medical care, aerospace, and packaging, etc [179]. Herein, we provide a summary about their thermal management applications. 

### 4.1. Electronic Devices

**Figure 7:**
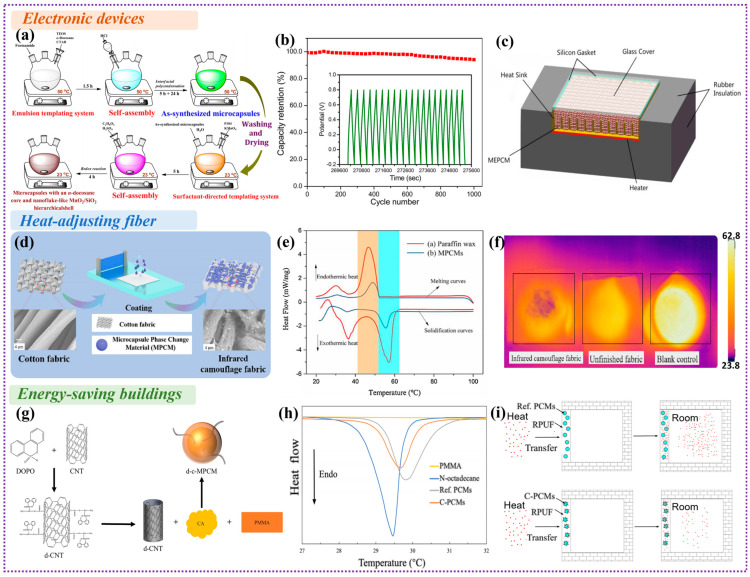
(**a**) Schematic diagram of synthesizing nanoflake−like MnO_2_/SiO_2_ hierarchical microcapsules containing n-docosane PCMs; (**b**) Plot of capacitance retention as a function of cycle number for microcapsules with a MnO_2_/SiO_2_ hierarchical shell. Reprinted with permission from [181]. Copyright 2018 Elsevier; (**c**) Schematic diagram of electronic device heat sink assembly. Reprinted with permission from [183]. Copyright 2020 Elsevier; (**d**) Preparation process and SEM image of infrared camouflage fabric; (**e**) DSC curves for the melting and solidification process of paraffin and paraffin/UF MEPCMs; (**f**) Infrared thermal imaging of infrared camouflage fabric, unfinished fabric, and blank control sample. Reprinted with permission from [184]. Copyright 2020 Elsevier; (**g**) Schematic diagram for preparing d-c-MPCM. Reprinted with permission from [185]. Copyright 2022 Elsevier; (**h**) DSC curves of PMMA, n-octadecane, PCMs, C-PCMs; (**i**) The process of heat transfer among PCMs and C-PCMs. Reprinted with permission from [186]. Copyright 2020 Elsevier.

Electronic devices will release large amounts of heat during operation, which may cause a dramatic rise of the temperature for electronic devices. If the heat dissipation problem was not addressed, it will severely affect the reliability and stability of the electronic devices or even cause irreversible damage. Fortunately, the heat storage and release characteristics of PCMs can absorb the heat of electronic devices during operation, thereby promoting the heat dissipation of the system and achieving promising temperature control [180]. For example, Xu et al. developed a n-docosane based MEPCMs with nanoflake-like MnO_2_/SiO_2_ hierarchical shell, and utilized as a thermoregulatory electrode material for in-situ thermal management of supercapacitors [181]. Figure 7a shows the schematic diagram of synthesizing nanoflake-like MnO_2_/SiO_2_ hierarchical microcapsules. The test results show that the MEPCMs presented a superior long-term cycling stability with high capacitance retention of 94.7% after 1000 charging/discharging cycles at 45 °C (Figure 7b). Besides, Wang et al. designed a novel MEPCMs by encapsulating paraffin core with CaCO_3_ shell, and investigated its morphology and temperature control strategy [182]. Compared with the original paraffin, the thermal conductivity of microcapsules is increased by 2.25–2.63 times due to the existence of CaCO_3_ shell. Ren et al. also prepared MEPCMs with paraffin as the core and CaCO_3_ as the shell material [183]. They further mixed it with expanded graphite (EG) to form a composite material, and the schematic diagram of PCM-EG composite based electronic device heat sink assembly was shown in Figure 7c. The thermal performance of electronic device is effectively enhanced by inserting MEPCM–EG composite. Up to now, the MEPCMs with various inorganic fillers are playing an increasingly important role in the thermal management field of electronic devices. Moreover, with the rapid development of low-dimensional thermally conductive nanofillers with synergetic electrical insulation, it will make the MEPCMs a superb candidate to achieve the highly efficient thermal management of electronic devices in more practical occasions.

### 4.2. Heat-Adjusting Fiber

Integrating PCMs into fibers offers exciting opportunities for smart textiles. The stored and released energy poised to make the human body more adaptable to external environments. For instance, Niu et al. synthesized a new type of wet-spun phase change fiber with outstanding flexibility, excellent electrical conductivity, high enthalpy, and adjustable phase temperature [187]. The enthalpy values of the fibers vary from 98.6 to 124.5 J/g according to the content of PCMs. The intelligent energy storage fiber with integrative properties could be woven into fabrics, providing a new option for smart textiles in wearable and protective systems. Besides, Wang et al. have developed a new smart textile based on PCMs by the incorporation of TiO_2_, which has UV shielding and temperature regulation functions [188]. The smart textile has a latent heat of 51.14 J/g and shows good thermal reliability after 500 heating-cooling cycles. Kizildag prepared polyacrylonitrile (PAN) nanofibers containing paraffin PCMs by electrospinning method [189]. By adding the PCMs with different phase transition temperatures into the nanofiber, the composite nanofibers with temperature regulation ability in a wider temperature range were obtained. Besides, the contact Angle measurements showed that the addition of PCMs made the nanofibers hydrophobic. Furthermore, thermal conductivity enhanced MEPCMs also have important applications in fibers and have attracted great attention in the textile industry. By adding MEPCMs into fiber and making full use of the excellent heat storage and release properties of PCMs, the intelligent textile with temperature regulation capability can be developed, which will guarantee the human body in a comfortable temperature range [190]. Nejman et al. add 20 wt% MEPCMs into the fabric to explore the influence of n-octadecane-based MEPCMs on the thermal properties and air permeability of fabrics [191]. It was found that the thermal properties and the permeability of fabric can be adjusted by the addition of MEPCMs. Moreover, to further improve the thermal comfort of textiles, Alay Aksoy et al. prepared a series of MEPCMs by emulsion polymerization using P(MMA-co-MAA) as the shell and n-octadecane as the core [192]. It was found that the average melting and freezing temperatures of the n-octadecane microcapsules were 27 °C and 26 °C, respectively, which confirmed the temperature regulating capabilities of MEPCMs on the fabric. Furthermore, Xu et al. developed paraffin based MEPCMs with a UF resin shell by in-situ polymerization and then coated on the fabric [184]. Compared with unprocessed fabrics, the fabrics containing MEPCMs can reduce the temperature by 5–10 °C and have a certain degree of infrared camouflage capability. The preparation process diagram and SEM images of infrared camouflage fabric are shown in Figure 7d. As depicted in Figure 7e, the DSC curves showed that the MEPCMs have a similar crystallization and melting process as the paraffin wax, and the shell material can prevent the thermal nucleation of core material. In addition, it can be clearly observed that the infrared camouflage fabric exhibits the smallest amount of infrared heat radiation and the darkest color from infrared thermal imager (Figure 7f), indicating that the as-prepared fabric by MEPCMs coating method has a superior thermal infrared camouflage effect. For developing multifunctional fabrics, the MEPCMs were also used to reduce ultraviolet radiation or achieve superhydrophobic function. For instance, Li et al. designed a new MEPCMs based on n-eicosane core and CuO doped polyurea shell by interfacial polymerization [193]. The thermal analysis showed that the MEPCMs had a high latent heat of 162.3 J/g, which achieved outstanding photothermal conversion ability and could reduce about 30% of UV radiation. Besides, the MEPCM has a water contact angle of more than 148°, which has excellent hydrophobic performance. It is foreseeable that more MEPCMs with various functions such as flame resistance, electromagnetic shielding, flexible sensing, will be reasonably designed to meet people’s higher pursuit of intelligent fabric. Particularly, low-dimensional nanofillers with many features including thermal conductivity, electromagnetic shielding and barrier effects will bring more opportunities for the development of advanced MEPCMs and their wider applications.

### 4.3. Energy-Saving Buildings

Nowadays, with the continuous development of economy and the gradual rise of carbon dioxide emissions, reducing building energy consumption has become an urgent problem to be solved. The researchers found that MEPCMs can be embedded in concrete, cement mortars and sandwich panels, which not only reduces energy consumption but also improves thermal comfort [194]. The selection of PCMs in buildings usually follows two principles: one is to choose the appropriate PCMs according to the range of indoor temperature change; the other is to choose non-toxic, non-corrosive, and low-cost PCMs. For instance, Cabeza et al. studied a new type of PCMs concrete, and the ultimate goal is to develop a product that can be used to improve the energy efficiency of buildings [195]. It is found that the internal temperature of the wall is reduced by adding MEPCMs, while their heat storage capacity is improved. Besides, Giro-Paloma et al. prepared paraffin MEPCMs with acrylic acid as shell [196]. The experiment found that the melting temperature of microcapsules was about 21 °C, which was suitable for building applications. Niu et al. develop a multifunctional MEPCMs with modified CNTs, which was added in rigid polyurethane foam (RPUF) to effectively improve the thermal comfort level of buildings (Figure 7g) [185]. In addition, Cheng et al. developed novel MEPCMs doped by CNTs (C-PCMs) [186]. DSC results exhibit that the latent heat of C-PCMs and PCMs are 112.25 J/g and 116.18 J/g, respectively (Figure 7h). The experimental results in the model chamber show that the composite filled with C-PCMs reduces indoor temperature fluctuation, and the thermal insulation ability of C-PCMs in RPUF is better than that of PMMA-coated MEPCMs, which shows the potential of improving building energy saving and thermal comfort (Figure 7i). 

### 4.4. Others

With the great advantages of efficient utilization of solar energy abundant in natural resources, the MEPCMs has become a very promising solar thermal conversion and energy storage material. Zhang et al. developed paraffin@SiO_2_/Ti_4_O_7_ MEPCMs by the sol-gel method, and the silica shell not only prevented the leakage of paraffin, but also improved its thermal conductivity [197]. The Ti_4_O_7_ nanoparticles achieve an effective absorption of sunlight and exhibit a high conversion from light to heat. Besides, Liu et al. designed and prepared a MEPCMs system based on N-eicosane core and CaCO_3_/Fe_3_O_4_ composite shell [198]. The MEPCMs synthesized under the optimal synthesis conditions have a potential heat storage capacity of about 140 J/g, a high encapsulation rate of more than 59%, and a high thermal conductivity of 0.795 W/(m·K). More importantly, due to the presence of nano-Fe_3_O_4_, the solar photothermal conversion and storage performance of the MEPCMs is significantly enhanced. The photothermal conversion efficiency of the MEPCMs is 47.9% higher than that of the corresponding microcapsule without Fe_3_O_4_, which has considerable potential in the efficient utilization of solar energy. In addition, MEPCMs also have potential applications in the fields of biomedicine. For instance, Zhang et al. synthesized a novel multifunctional N-epoxide based microcapsule with silver/silica bilayer shell by interfacial polycondensation [199]. The synthesized MEPCMs have regular spheres, high potential heat storage, high release efficiency and excellent heat regulation ability. More importantly, these microcapsules showed outstanding antibacterial activity against Staphylococcus aureus and Bacillus subtilis, and the bactericidal rate reached more than 95% after 4 h exposure. Furthermore, a new desalination method using MEPCMs and thin film evaporation was proposed [200]. In this process, the MEPCMs is placed in ocean water, and then the seawater and MEPCMs are ejected into a vacuum flash chamber. A thin film of seawater liquid is formed on the surface of MEPCMs, which was subsequently evaporated. The results show that MEPCMs significantly increases evaporative heat transfer, improves desalination efficiency, and reduces seawater corrosion. In addition to the applications mentioned above, MEPCMs, as a promising energy storage medium, can be further expanded to other thermal management fields through reasonable structural design and functionalization in the future.

## 5. Conclusions and Future Perspectives

PCMs are a particularly attractive TES material to address the mismatch between energy supply and demand in time and space. Nevertheless, most pristine PCMs suffer from some undesired problems such as leakage, low thermal conductivity, phase separation, supercooling, and corrosion, which seriously hinder their widespread practical application. To overcome aforementioned bottlenecks, enormous efforts have been dedicated to constructing various advanced MEPCMs and exploring their potential applications. In particular, the MEPCMs reinforced with low-dimensional nanofillers have sparked extensive interest of researchers during the past few years. In this review, we strive to provide a comprehensive and profound understanding of the recent advances, emerging trends, and challenges of MEPCMs with various low-dimensional thermally conductive fillers. We first discuss the typical PCMs, shell materials, and preparation strategies for constructing advanced MEPCMs. After that, we summarize the influence of low-dimensional nanofillers on thermal conductivity of MEPCMs as well as their thermal management application. Notably, although low-dimensional nanofillers can significantly improve the properties of MEPCMs and endow the MEPCMs with special function, in our opinion, several scientific issues and technical difficulties remain to be resolved prior to commercial uses:

(1) The incorporation of low-dimensional thermally conductive nanofillers in MEPCMs can indeed effectively enhance the thermal conductivity of PCMs, which is critically significant to promote their thermal charging and discharging in practical thermal management systems. However, a quantitative theory between low-dimensional nanofillers and thermal conductivity is still lacking. Moreover, it is also challenging to achieve the uniform dispersion of nanofillers in MEPCMs, and the resultant high interfacial thermal resistance causes the superiority of low-dimensional nanofillers have not been fully exploited. In addition, the energy storage density of MEPCMs is usually lower than that of pristine PCMs in many cases. It is challenging to achieve a promising balance between high energy storage density, low leakage, superior thermal conductivity, and highly efficient energy conversion. It is highly advisable to explore some novel strategy to optimize the interfaces and aligned heat transfer networks.
(2) Although the MEPCMs reinforced with low-dimensional nanofillers exhibit many impressive properties, their large-scale production is still challenging due to the complicated manufacturing process and high costs. For example, BNNS, with high in-plane thermal conductivity, distinctive chemical inertness, and excellent inherent electrical insulation, is very suitable for developing advanced MEPCMs, which exhibits promising applications in electronic devices. However, it is difficult and time-cost to obtain high-quality and defect-free BN nanosheets with large lateral sizes. Besides, as for the encapsulation strategies, simple and feasible synthetic methods are worthy exploring by high-throughput simulation and machine learning. Moreover, with the increasing environmental awareness, the eco-friendly raw materials and solvent-free synthesis also become crucial.
(3) Versatile MEPCMs for multi-purpose thermal management applications should be investigated. Until now, most research focus of MEPCMs containing low-dimensional nanofillers was placed on achieving synergetic enhancement of higher thermal conductivity, efficient energy conversion, and larger energy storage density, while their cycle stability, supercooling, and phase separation problems are usually ignored. Besides, the other functions of MEPCMs such as humidity control, flame resistance, electromagnetic shielding, flexible sensing should also receive adequate attentions for satisfying the specific requirements of their widespread applications in the future. Moreover, a database on MEPCMs with low-dimensional nanofillers should be established for better design of MEPCMs-based thermal management systems with targeted thermal performance even though there is still a long way to achieve this goal. In summary, by systematically summarizing the relevant achievements and exploring their preparation–structure–properties-applications relationships, we hope this review provide in-depth insights and meaningful enlightenment for the architectural design and multifunction of advanced MEPCMs.

## Figures and Tables

**Table 1:** Advantages, disadvantages and melting points of typical PCMs.

Type	PCMs	Advantages	Disadvantages	Melting Point (°C)
Organic	Paraffin C16-C18	Wide applicable temperature range Less supercooling phenomenon High crystallization rate High chemical stability Strong recycling performance Safety and non-toxic Little corrosion performance	Low thermal conductivity Poor thermal storage capacity per unit volume Volatilize easily Flammability Expensive	22
	Paraffin C20-C33			50
	Stearic acid			69
	Palmitic acid			56
	Myristic acid			58
	Lauric acid			49
Inorganic	MgSO_4_·7H_2_O	Lower volumetric expansion Large heat storage capacity Low cost High thermal conductivity Greater phase change enthalpy Non-flammable With a clear melting point Recyclable	High supercooling Prone to precipitation Poor dimensional stability Low thermal stability Corrosive Phase segregation Poor compatibility with some building materials	49
	CaCl_2_·6H_2_O			33
	Ba(OH)_2_·8H_2_O			78
	Al(OH_3_)_2_·7H_2_O			75
	KNO_3_			340
	Na_2_CO_3_			850
Eutectic	CO(NH_2_)_2_-NH_4_Br	High thermal conductivity Hardly segregation Hardly supercooling	Expensive Strong odor	80
	Na(CH_3_COO)·3H_2_O-CO(NH_2_)_2_			30
	NaCO_3_-LiCO_3_			490
	NaF-MgF_2_			640

**Table 2:** Typical shell materials in the preparation of MEPCMs.

Shell Type	Shell Materials	Core Materials	Encapsulation Methods	Phase Change Enthalpy (J/g)	Encapsulation Rate (%)	Refs.
Organic shells	MF	Lauryl alcohol	In-situ polymerization	187.5	93.1	[45]
	UF	Paraffins	In-situ polymerization	72.4	52.8	[49]
	PU	Methyl laurate	Interfacial polymerization	136.2	-	[63]
	PMMA	Na_2_HPO_4_·12H_2_O	Solvent evaporation	142.9	80.4	[58]
	Polyurea	CaCl_2_·6H_2_O	Interfacial polymerization	118.3	71	[60]
	PS	N-tetradecane	In-situ polymerization	98.71	44.7	[70]
	Starch	N-heptadecane	Interfacial polymerization	187.27	78.27	[71]
Inorganic shells	SiO_2_	N-octadecane	Interfacial polymerization	109.5	51.5	[73]
		Mannitol	Sol-gel	252.66	89.6	[86]
	CaCO_3_	N-tetradecane	Self-assembly	58.54	25.86	[91]
	rGO	SA	Pickering emulsion polymerization	159	74.3	[96]
	TiO_2_	N-eicosane	Interfacial polymerization	188.27	-	[99]
Organic-inorganic hybrid shells	MF-SiO_2_	N-hexadecanol	Pickering emulsion polymerization	163.76	74.6	[103]
	PMMA-BN/TiO_2_	Paraffin	Pickering emulsion polymerization	124.4	72.1	[104]
	P(MMA-co-BA)-TiO_2_	Paraffin	Pickering emulsion polymerization	90.12	36.09	[105]
	PUA-TiO_2_	N-octadecane	Interfacial polymerization	181.1	77.3	[106]
	PS-GO	N-hexadecane	Pickering emulsion polymerization	186.8	78.5	[108]

**Table 3:** Comparison and summary of the different preparation methods of MEPCMs.

Types	Methods	Advantages	Disadvantages	Scope of Applications
Physical method	Spray drying	Simple operation High production efficiency Wide range of sizes	Low packaging rate Not used for inorganic PCMs	Organic PCMs Heat sensitive material
	Solvent evaporation	Low cost	Low encapsulation efficiency	Inorganic PCMs
Chemical method	In-situ polymerization	High encapsulation efficiencyStable shape Uniform coating	Complex operation Harmful for the environment	Organic PCMs Inorganic PCMs Organic shell material such as MF and UF
	Interfacial polymerization	High reaction speed Simple operation Low permeability	The monomer is required to have a high reactivity Harmful for the environment	Organic PCMs Inorganic PCMs Organic shell material such as UF
	Suspension polymerization	Environmentally friendly Facile reaction condition High packaging rate	High energy consumption Expensive Not used for inorganic PCMs	Organic PCMs Large grained Organic shell material such as PMMA
	Emulsion polymerization	Stable High packaging rate High preparation efficiency Environmentally friendly	Complicated Expensive	Organic PCMs Inorganic PCMs Organic shell material such as PMMA Polymer/inorganic hybrid shell
Physical-chemical method	Coacervation	Simple equipment Good control of the particle size and thickness	Difficult to scale-up Not used for inorganic PCMs Agglomeration	Organic PCMs Organic shell material
	Sol-gel method	High thermal stability Strong controllability	Non-insulated and limited in building applications	Organic PCMs Inorganic PCMs Inorganic shell material

**Table 4:** Summary of the thermal conductivity of various MEPCMs with low-dimensional nanofillers.

Dimensional	Thermally Conductive Fillers		MEPCMs	Thermal Conductivity (W/(m·K))	Refs.
Zero-dimensional	Graphite nanoparticles		Paraffin@MF/graphite nanoparticles	0.312	[134]
	Copper nanoparticles		Hexadecane@Copper nanoparticles interlocking polydivinylbenzene	0.5045	[135]
	SiC nanoparticles		N-octadecane@PMF/SiC	0.21	[136]
	Ag nanoparticles		N-OD@Silica/PDV/Ag	1.346	[137]
	Fe_3_O_4_ nanoparticles		Paraffin@Fe_3_O_4_ nanoparticles	-	[138]
One-dimensional	CNTs		Paraffin@MWCNTs	0.355	[140]
			N-docosane@SiO_2_/PANi/CNTs	0.846	[141]
			C18/C28@MF/OI modified CNTs	0.329	[142]
	AgNWs		1-Tetradecanol@AgNWs	1.46	[144]
Two-dimensional	Graphene		Paraffin@GNPs	0.378	[140]
			N-hexadecane@PS/GO	-	[108]
			SA@Graphene	0.352	[155]
			Paraffin@GO/PbWO_4_	0.735	[156]
			Dodecanol@MF/GO	0.2790	[157]
	BN		Paraffin@SiO_2_/BN	0.675	[167]
			N-octadecane@BN/MF	0.184	[12]
			SA@SiO_2_/m-BN	0.506	[169]
	BPs		Eicosane@PMMA/mBPs	-	[13]
Low-dimensional hybrids	“Point-line” structure	Cu_2_O/Cu/MWCNTs	Paraffin@Cu_2_O/Cu/MWCNTs	0.43	[173]
		Graphite nanoparticles/CNTs	Sodium Thiosulfate Pentahydrate@CNTs/Graphite nanoparticles	4.031	[174]
	“Point-plane” structure	TiO_2_ nanoparticles/BN	Paraffi@PMMA/BN/TiO_2_	0.4215	[104]
		AgNPs/2D-Mt	SA@2D-Mt/AgNPs	0.8223	[177]
		SiO_2_/GO	Paraffin@SiO_2_/GO	1.162	[178]
	“Line-plane” structure	CNTs/GO	Dodecanol@MF/GO/CNTs	0.3821	[175]
		MWCNTs/GNPs	Paraffin@MWCNTs/GNPs	0.430	[140]

## Data Availability

Not applicable.
